# Transcranial pulse stimulation to treat multiple system atrophy: a case report

**DOI:** 10.1007/s00415-025-13503-4

**Published:** 2025-11-10

**Authors:** Johannes Stalter, Karsten Witt

**Affiliations:** 1 Department of Neurology, Carl Von Ossietzky University Oldenburg, Oldenburg, Germany; 2 University Clinic of Neurology, Evangelical Hospital Oldenburg, Oldenburg, Germany; 3 Research Center Neurosensory Science, University of Oldenburg, Oldenburg, Germany

Dear Sirs,

Multiple System Atrophy of the parkinsonian subtype (MSA-P) is a neurodegenerative disease which affects not only the motor system but also autonomic, affective and cognitive functions. The treatment so far is only symptomatic, including probatory levodopa treatment, occupational training, and physical therapy. Motor symptoms mainly consist of Parkinsonism, i.e., rigidity, bradykinesia, and tremor in addition to cerebellar ataxia. In some cases, levodopa treatment shows to be effective for these symptoms. Nevertheless, often times no significant treatment success can be achieved with drug treatment, especially when looking at the long-term outcomes. [1]

TPS is a non-invasive neuromodulation technique utilizing focused ultrasound waves to enhance brain function, with real-time visualization of pulse application [2, 3]. Studies in Alzheimer’s and Parkinson’s disease have demonstrated its safety and efficacy. In this case report, we extended these findings from case series into a new disease model, Multiple System Atrophy.

The patient we present is a 73-year-old male with a 3-year history of clinically confirmed MSA-P using the MDS-MSA diagnostic criteria at the time-point TPS therapy started. Retrospectively, first symptoms were evident 5 years before TPS treatment, while drug therapy was introduced three years before TPS treatment at the time-point of the MSA diagnosis.

The leading symptoms are rigidity and bradykinesia in combination with a marked postural instability and extensive hypotension, leading to recurrent falls. Due to the latter two, the patient is wheelchair-bound. Activities of daily living are only possible with full help of caregivers. Besides MSA, a restless-legs-syndrome, a deep vein thrombosis as well as chronic anemia due to iron deficiency are known. Following the MSA diagnosis, the patient showed a slight but beneficial response to levodopa. As a result, therapy involving piribedil (200 mg per day), levodopa (600 mg per day), levodopa extended-release 100 mg daily, as well as physical and occupational therapy was initiated and well tolerated. This pharmacological treatment was introduced one year before TPS therapy began and was continued throughout the entire TPS treatment period. Additionally, apixaban 5 mg twice a day, clonazepam 0.5 mg, and tamsulosin 0.4 mg were taken once a day. Disease-specific drugs like levodopa or dopamine agonists showed a mild but for the patient noticeably improvement of the motor symptoms. Since the disease is progressive, a constant worsening of the patient’s status could be observed over the disease course. To address this worsening, other treatment options were discussed with the patient.

After a detailed risk explanation, informed written consent, and the exclusion of cerebrovascular abnormalities in the MRI as a contraindication, TPS was administered. As described in the study by Osou et al. for Parkinson patients, we targeted the prefrontal cortex, primary motor cortex, and supplementary motor area [4]. Over a period of 2 weeks, in total, ten sessions took place. Each session delivered 4000 pulses at 0.25 mJ/mm^2^ and 4 Hz evenly distributed over the targeted areas, as inspected by the TPS visualization tool (Fig. 1).

**Fig. 1:**
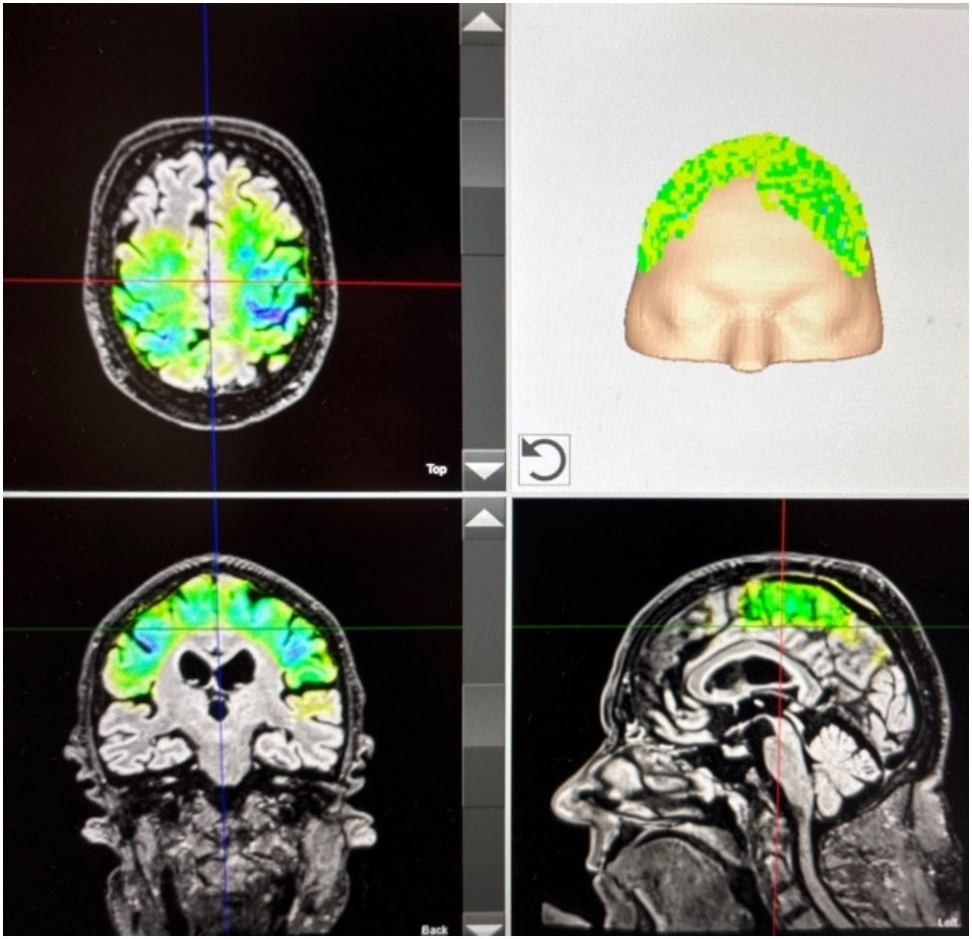
Example of stimulation visualization during a session. Individual neuro-navigation is calculated before each session for visualization purposes. The color indicates the calculated energy applied in the respective area. The darker the color, the more energy was delivered. The location of the delivered impulses is shown in the upper right quadrant

Consecutively, six sessions were administered over 1 year, resulting in a total of 16 sessions over the period of 1 year. The sessions were performed by a trained and in TPS-experienced neurologist using the NEUROLITH TPS system (Storz Medical AG, Tägerwilen, Switzerland). Medication and physical therapy remained unchanged during the TPS therapy. Motor symptoms were assessed using the Movement Disorder Society-Unified Parkinson's Disease Rating Scale (MDS-UPDRS) part III and the Unified Multiple System Atrophy Rating Scale (UMSARS), part I and II. Both scales were examined before the first block, after ten sessions and after 1 year. Treatment side effects were recorded at the same time points. Patient-reported outcomes and caregiver feedback were also recorded after the initial block and one year. This study been performed in accordance with the ethical standards laid down in the 1964 Declaration of Helsinki and its later amendments.

Post-treatment, there was a 22.2% improvement in the total MDS-UPDRS score, with main reductions in rigidity (improvement of 50%) and bradykinesia (improvement of 26.9%). Axial symptoms remained largely unchanged (improvement of 5%). Regarding the subscores differentiating between the region of improvement, the extremities exhibited the largest improvement with 36% over the initial block and 47% over the whole year, the improvement between initial block and the follow-up was still 17%. Axial symptoms improved 9% after the initial block and remained unchanged during the following year. Gait did not improve by TPS therapy. All relative and absolute changes are reported in Table 1.

**Table 1:** Changes in the MDS-UPDRS and the UMSARS

MDS-UPDRS, part III			
	Extremities	Axial	Gait
Pre	36	11	8
Post	23	10	8
FU	19	10	8
Pre–post	− 36% (13)	− 9% (1)	0%
Pre-FU	− 47% (26)	− 9% (1)	0%
Post-FU	− 17% (4)	0%	0%

	Total	Rigidity	Bradykinesia	Axial
Pre	63	12	26	19
Post	49	6	19	18
FU	44	3	17	18
Pre–post	− 22% (14)	− 50% (6)	− 27% (7)	− 5% (1)
Pre-FU	− 30% (19)	− 75% (9)	− 35% (9)	− 5% (1)
Post-FU	10% (5)	50% (3)	10% (2)	0%

UMSARS, part I and II		
	Motor	Autonomic
Pre	38	32
Post	25	27
FU	33	30
Pre–post	− 34% (13)	− 16% (5)
Pre-FU	− 13% (5)	− 6% (2)
Post-FU	32% (− 8)	11% (− 3)

This table shows the absolute values of the different sub-scores for the MDS-UPDRS III and the UMSAR part I (motor functioning) and II (autonomic function). Additionally, the change in each sub score is given as the relative change and the absolute change (in brackets). *Pre* baseline before initial stimulation block, *post* after initial stimulation block, *FU* (Follow up) = after 1 year, *MDS-UPDRS* Movement Disorder Society-Unified Parkinson’s Disease Rating Scale, *UMSARS* Unified Multiple System Atrophy Rating Scale

UMSARS motor scores during the initial block improved by 34%. After 1 year, there was still an improvement of 13% compared to the initial status. During the 1-year period after the initial block, the motor symptoms worsened by 32%, but stayed below the initial level (Table 1). The autonomic symptoms first improved by 15% during the initial block, the total improvement over 1 year was only 6%. After the initial block, the symptoms got worse by 11%. Patient and caregivers reported enhanced upper limb function and daily living activities after the initial block but no benefit over the 1-year period. The patient experienced transient, mild sensory discomfort during stimulation but no other adverse effects.

In the reported case, we showed that an initial block of TPS sessions could considerably improve the motor symptoms but had no effects on the autonomic dysfunctions. After receiving TPS therapy, the MDS-UPDRS showed marked improvement of the motor functions, especially of the extremities with rigidity and bradykinesia, while pharmacotherapy remained constant. The results could partially be maintained over the period of 1 year. Transient and mild adverse side effects were reported. Caregivers reported an improvement also in the daily functioning, e.g., personal hygiene and dressing. This combination could lead to a promising approach to improve symptoms in a disease where treatment options are limited. To the best of our knowledge, there are no studies testing this treatment approach in MSA.

Previous studies using TPS to modulate brain functions used functional magnetic resonance imaging (fMRI) or sensory-evoked potentials (SEPs) proved the ability of TPS to modulate neuronal activity [3, 5]. First, an increasing number of pulses to the primary sensory cortex lead to an increase in the SEP amplitude of median nerve stimulation in a dose-dependent manner, demonstrating higher cortical excitability following TPS administration. Second, TPS were able to upregulate network activity and increased functional connectivity of the stimulated areas in Alzheimer’s dementia patient [5]. This is thought to work via mechanical effects on mechanosensitive ion channels with a consecutive change in neuro-transmitter distribution and network changes. Since the pathomechanisms of PD and MSA-P are quite different, i.e., PD as a pre-synaptic disease and MSA-P as a post-synaptic one, the rationale to apply TPS in MSA was based on stimulating the post-synaptic network affected by MSA [6]. This is supported by the finding of an increased functional connectivity as TPS may increase the connectivity of cortical–subcortical motor areas and therefore enable supporting cortical motor networks to mitigate malfunctioning of basal ganglia loops. As a potential long-term effect, this could lead to an increased neuronal plasticity.

Clinical data in Parkinson’s disease (PD) showed an improvement of motor functions using the same stimulation protocol as in this report [4]. The target regions in this study as well as in the PD study were chosen to stimulate the motor system in general, to enhance neural networks involved in these areas. This might explain why there were no clear changes in autonomic dysfunctions or the postural instability of the patient. The effects for some domains were permanent over 1 year, with some even improving under the maintenance therapy in a disease with a usually progressive course. This progressive nature of the disease could be one part of an explanation why most effects diminished over the whole year. Another aspect of a potential explanation could be the dose–effect correlation observed for SEPs modulated with TPS, as the sessions frequency during the year was lower than in the initial block [2, 3]. Those two aspects should be investigated together with the potential long-term effects of neuronal plasticity and its changes caused by TPS. Potential strategies to maintain these effects could be a higher frequency of booster sessions or personalized treatment protocols using the neuro-navigation and symptom-focused target regions.

Taken together, TPS was able to improve motor symptoms in a patient that showed only a noticeable but not sufficient reaction to standard drug treatment.

Clearly, one has to keep in mind that this is an uncontrolled case report, so the potential placebo effect may play an important role in the results. The study by Osou et al. lists several reasons which make a placebo effect likely in this TPS protocol; all of those reasons apply for this case report as well [4]. In PD, it is known that the expected treatment effect is causing a dopamine release which in turn improves the dopamine-depending symptoms and can account for up to 55% of the observed effect. Since MSA-P is caused by a post-synaptic dysfunction, the effect size of placebo in the reported patient should be smaller.

Nevertheless, this case suggests that TPS may offer therapeutic benefits for MSA-P, particularly in improving upper limb motor function. Further research, including blinded studies, is necessary to evaluate the safety, efficacy, and potential placebo effects of TPS in MSA-P patients. This can be extended by including different target regions, e.g., the cerebellum which plays an important role in MSA, to treat atactic symptoms and adapt the therapy better to the individual symptom burden. Furthermore, the underlying mechanism of action should be investigated in basic research trials, e.g., electrophysiological changes in short intracortical inhibition and intracortical facilitation. Finally, the aspects regarding the long-term effects directly open new research questions, which should be further examined. This would also help to understand potential long-term effects of this treatment option.

## Data Availability

The data that support the findings of this study are available from the corresponding author, JS, upon reasonable request.

## References

[1] Poewe W, Stankovic I, Halliday G et al (2022) Multiple system atrophy. Nat Rev Dis Prim 8(1):1–21. 10.1038/s41572-022-00382-6 10.1038/s41572-022-00382-6 36008429

[2] Beisteiner R, Hallett M, Lozano AM (2023) Ultrasound neuromodulation as a new brain therapy. Adv Sci 10(14):2205634. 10.1002/ADVS.202205634 10.1002/advs.202205634 PMC10190662 36961104

[3] Beisteiner R, Lozano AM (2020) Transcranial ultrasound innovations ready for broad clinical application. Adv Sci 7(23):2002026. 10.1002/ADVS.202002026 10.1002/advs.202002026 PMC7709976 33304757

[4] Osou S, Radjenovic S, Bender L et al (2024) Novel ultrasound neuromodulation therapy with transcranial pulse stimulation (TPS) in Parkinson’s disease: a first retrospective analysis. J Neurol 271(3):1462–1468. 10.1007/S00415-023-12114-1 38032371 10.1007/s00415-023-12114-1 PMC10896933

[5] Beisteiner R, Matt E, Fan C et al (2020) Transcranial pulse stimulation with ultrasound in Alzheimer’s disease—a new navigated focal brain therapy. Adv Sci 7(3):1902583. 10.1002/ADVS.201902583 10.1002/advs.201902583 PMC7001626 32042569

[6] Kaasinen V, Kankare T, Joutsa J, Vahlberg T (2019) Presynaptic striatal dopaminergic function in atypical Parkinsonism: a metaanalysis of imaging studies. J Nucl Med 60(12):1757. 10.2967/JNUMED.119.227140 30979821 10.2967/jnumed.119.227140 PMC6894374

